# Determining the effect of exogenous organic materials on spatial distribution of maize yield

**DOI:** 10.1038/s41598-019-56266-5

**Published:** 2019-12-27

**Authors:** Bogusław Usowicz, Jerzy Lipiec

**Affiliations:** Institute of Agrophysics, Polish Academy of Sciences, ul. Doświadczalna 4, 20-290 Lublin, Poland

**Keywords:** Ecology, Physics

## Abstract

Knowledge on spatial distribution of crop yield in relation to fixed soil fertilisation with exogenous organic materials is essential for improving precise crop and soil management practices within a field. This study assessed the effect of various application rates and types of exogenous (recycled) organic materials (EOMs) containing different organic matter and nitrogen contents vs. mineral nitrogen on the yield of maize by means of linear regressions (trends), spatial kriging-interpolated maps, and Bland-Altman statistics. The experiments were conducted in 2013 and 2014 on two soils, i.e. loam silt in Braszowice (Poland) and clay silt loam in Pusté Jakartice (Czech Republic) under a cross-border cooperation project. The organic materials included compost from manure, slurry, and straw (Ag), industrial organic compost from sewage sludge (Ra), animal meal from animal by-products (Mb), and digestate from a biogas fries factory (Dg). The following 3 application rates of each EOM were adjusted according to the reference 100% = 200 kg N ha^−1^: 50 (50% N from EOM and 50% mineral N), 75 (75% N from EOM and 25% mineral N), and 100 (100% N from EOM). 100% mineral N was applied on control plots. All treatments were carried out in 4 replicates. The linear regressions between the EOM application rates and the maize yield were in general ascending in the Braszowice soil and descending in the more productive Pusté Jakartice soil. The spatial kriging-interpolated maps allowed separating zones of lower and higher yields with EOMs compared to the control. They were attributed in part to the different EOM application rates and soil water contents. The Bland-Altaman statistics showed that addition of 50% of N from EOMs in 2013 caused a decrease and an increase in the maize grain yield in Braszowice and Pusté Jakartice, respectively, whereas the inverse was true with the 75 and 100% EOM additions. In 2014, the yield of maize for silage increased with the increasing EOM application rate in Braszowice and decreased in Pusté Jakartice, but it was smaller on all EOM-amended plots than in the control. As shown by the limits of agreement lines, the maize yields were more even in Pusté Jakartice than Braszowice. These results provide helpful information for selection of the most yield-producing EOM rates depending on the site soil conditions and prevalent weather conditions.

## Introduction

Soil organic matter (SOM) is a key factor affecting many ecosystem services including nutrient cycling that support crop production worldwide^1–4^. However, its content in soil decreases due to land-use change from natural forest or perennial grasslands to cultivated croplands; then, it is frequently subjected to intensive soil disturbance by conventional tillage practices^3,5,6^. It was reported that about 42 to 78 Gt of carbon were lost globally^7^ due to tillage and narrow crop rotation without legumes and cover crops and enhanced biological decay and soil erosion^8,9^. The loss of SOM from the topsoil of 20 cm due to soil disturbance and inadequate return of organic matter within a 30–50-year time period has been assessed as high as 50%^10^. The rate of SOM loss is increasing with progressive warming related to climate change^3,11^. The total rate of biomass and SOM losses is estimated to be up to 20% of worldwide atmospheric carbon dioxide emissions^12,13^. Therefore, the decline in SOM is considered as an environmental threat and a soil degradation component, as specified in the Communication from the European Commission “Thematic Strategy for Soil Protection”^14^.

To avoid further reductions and reverse the current SOM content, an international research *4 per mille* initiative has been launched at the Conference of the Parties (COP21) in Paris meeting (2015), which requests to increase carbon stocks at 4 per 1000 (or 0.4%) per year on agricultural lands to mitigate the SOM losses caused by climate change and to increase food production for the rising world population while being environmentally sustainable^9,15,16^. This initiative is supported by the fact that the quality and productivity of the soil resources of the Earth are deteriorating^2^.

One ecological management approach recommended to offset loss of SOM content and crop productivity is site-specific application of exogenous (recycled) organic materials containing organic carbon and nutrients^4,17,18^. Such materials can originate from waste products of agricultural, livestock, and biogas production systems. The use of organic materials can allow farmers to diminish waste streams and application of less chemical input-dependent and thus more sustainable soil management reducing reliance on high-price mineral fertilisers and pesticides in crop production^4,19,20^.

Recent research based on meta-analysis of numerous experiments^21^ showed that finding the direct effect of organic inputs on crop yields is difficult because of the additional effect on some environmental factors (e.g. flourishing soil biota). The authors suggested more in-depth research to describe the crop yield response to organic inputs more comprehensively. To separate direct effect of a given treatment the statistical Bland-Altman method can be adopted. In this approach the differences between two treatments or methods are plotted against their averages. The ensuing diagram allows determining average difference (bias), confidence of intervals for the bias and limits of agreement that indicate how much the new treatment or method differ from the old. This approach is used in biostatistics, medicine (e.g.^22^) and also in satellite studies^23,24^.

The aim of the work was (i) to determine the spatial variability and distribution of maize yield on soil fertilised randomly with spatially different amounts of exogenous organic matter, (ii) to examine the effect of different percentages of nitrogen from exogenous organic materials vs. conventional mineral nitrogen fertiliser only on the maize yield using Bland-Altman statistics, and (iii) to identify areas for improving the maize yield with consideration of site conditions. It was hypothesised that the Bland-Altman statistics distinguishes the effect of different portions of nitrogen from exogenous organic materials on the maize yield.

## Materials and Methods

**Figure 1:**
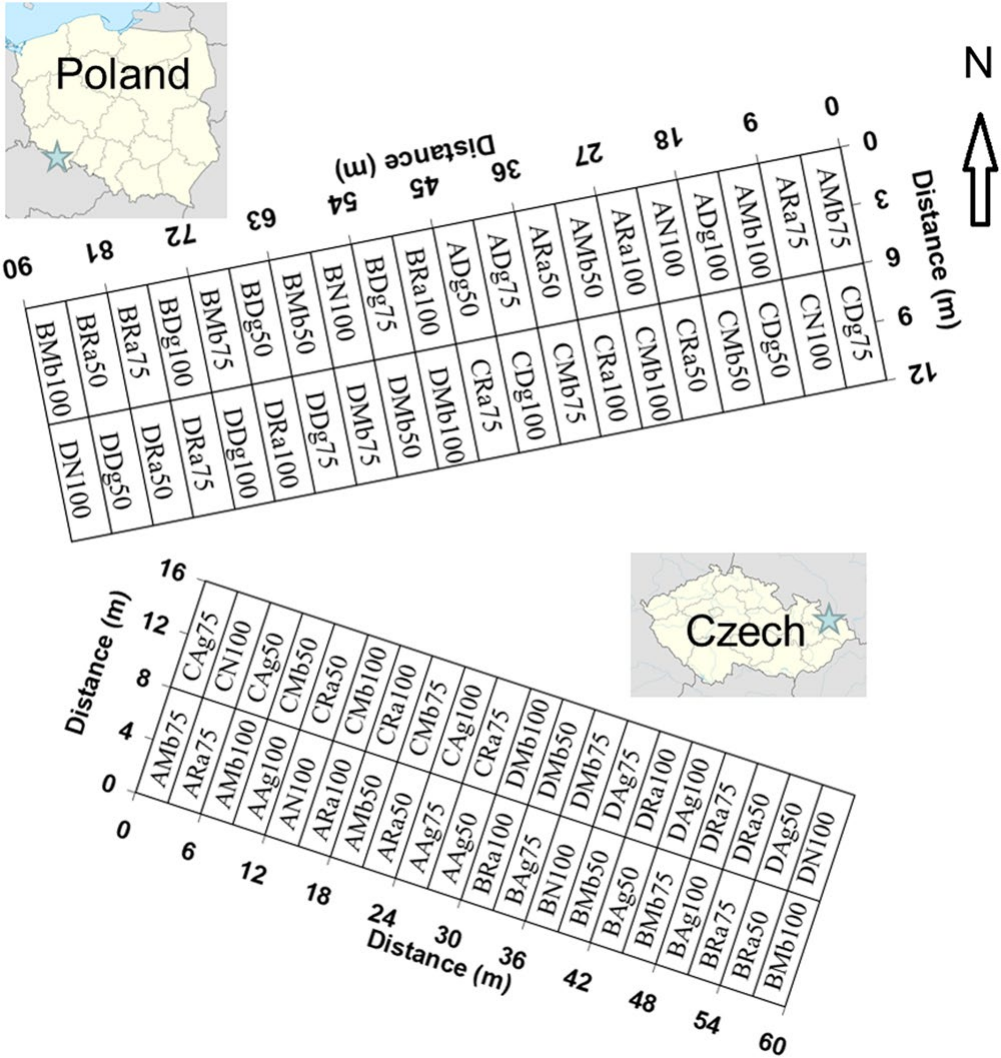
Layout of the experimental plots at the research sites in Braszowice (Poland) and Pusté Jakartice (Czech Republic). Explanations: initial capital letters A, B, C, and D refer to blocks (replicates) of the experiment. 50 = 50% N from a given EOM and 50% mineral N, 75 = 75% N from a given EOM and 25% mineral N, and 100 = 100% N from EOM, Control = 100% mineral N. Ag = compost from manure, slurry, and straw, Ra = industrial organic compost from sewage sludge, Mb = animal meal from animal by-products, Dg = digestate from the biogas fries factory. The maps of Poland and Czech are from wikipedia.org https://pl.wikipedia.org/wiki/Plik:Poland_location_map_white.svg. Author Poland location map.svg: NordNordWest (https://commons.wikimedia.org/wiki/User:NordNordWest). derivative work: Mareklug (https://commons.wikimedia.org/wiki/User:Mareklug). Licence CC BY SA 3.0: https://creativecommons.org/licenses/by-sa/3.0/. Author NordNordWest (https://commons.wikimedia.org/wiki/User:NordNordWest). Licence CC BY SA 3.0: https://creativecommons.org/licenses/by-sa/3.0/.

A field experiment was carried out on loamy silt soil in Braszowice, Poland (50° 34′ 03.0″N 16° 48′ 07.4″E), and clay silt loam in Pusté Jakartice, Czech Republic (49° 58′ 23.5″N 17° 57′ 19.8″E), in 2013 and 2014 under a cross-border cooperation project^25,26^. The soils contain 14 and 20% of clay and 1.02 and 1.13% of organic carbon, respectively. Both field sites are located about 140 km apart from each other. The experimental field in each site received 10 treatments, i.e. 3 exogenous organic materials (EOMs) × 3 rates and the control, with digestate from a biogas fries factory (Dg) in Braszowice, compost from manure, slurry, and straw (Ag) in Pusté Jakartice, and industrial organic compost from sewage sludge (Ra) and animal meal from animal by-products (Mb) in both fields. The following 3 application rates of each EOM were adjusted according to the reference 100% = 200 kg N ha^−1^: 50 (50% N from EOM and 50% mineral N), 75 (75% N from EOM and 25% mineral N), and 100 (100% N from EOM). 100% mineral N was applied on the control plots. The treatments were replicated in 4 blocks (A, B, C, D), which gave forty plots. Each plot had a surface area of 20 m^2^ in Braszowice and 25 m^2^ in Pusté Jakartice. The layout of the experments is shown in Fig. 1. The total nitrogen contents were 6.9, 2.6, 2.3, and 8.4% in Dg, Ag, Ra, and Mb, respectively. The corresponding organic carbon contents were 40.7, 24.1, 17.9, and 40.1%. The Mb, Ra, and Ag were applied twice in Pusté Jakartice. Ra and Dg were applied twice and Mb once in Braszowice. Instead of phosphorus-containing Mb, a mineral N fertiliser was applied in the second study year in Braszowice to avoid the possible risk of eutrophication.

A conventional tillage system including moldboard ploughing to a 25-cm depth was applied in both sites in late autumn, followed by a cultivator (15 cm) and pre-sowing surface preparation with a harrow in spring to prepare the seedbed. The EOMs were applied on the soil surface and then mixed into a 15-cm depth by a cultivator before seedbed preparation and sowing maize (*Zea mays* L.). The variety N K Terada, FAO 260 was grown in 2013 for grain and Ulan-FAO 270 was cultivated in 2014 for silage in both sites. On each plot, maize was grown in four rows and six rows in Braszowice and Pusté Jakartice, respectively. The maize was harvested by hand cutting at a height of 10 cm from two central rows to determine the grain and straw yield in 2013 and the silage yield in 2014^27^.

Basic statistics including the average, standard deviation, coefficient of variation, minimum and maximum values, skewness, kurtosis, and linear regressions between the EOM application rates and the yield components were calculated. Spatial dependence and distribution of the yield components were evaluated using geostatistical methods. Experimental semivariograms were computed and then mathematical functions were adjusted to semivariograms that were used for mapping by ordinary kriging^28,29^.

In order to determine a separate effect of the different application rates of N from the EOMs vs. control plots (only mineral nitrogen fertiliser) on the maize yield, the Bland-Altman statistics was adopted^22,30^. In this graphical method, the differences in the maize yields between the plots with different EOM nitrogen application rates and the control plots against the average yield with and without EOMs were determined. The agreement between the yield in the EOM-amended and control plots was assessed using bias (average of differences between the yields from the EOM-amended and control plots), the limit of agreement (LoA) defined as bias ± 1.96 × standard deviation, confidence intervals (CI) for the bias and LoA defined as ± standard error × the value of t distribution with n–1 degrees of freedom, and the Bland-Altman ratio (BAR) defined as the ratio of half the range of LoA to the mean of the pair including the yield from the EOM-amended and mineral nitrogen fertilized plots. The agreements were graded as good, moderate, and insufficient for BAR values < 0.1, 0.1–0.2, and >0.2, respectively^22^. Root mean square residuals (RMSR) and maximum relative residuals (MRR), which are the difference in the yield between the EOM-amended and control plots were determined for all yield components in 2013 (grain, straw, and grain plus straw) and 2014 (silage yield).

### Weather conditions

**Figure 2:**
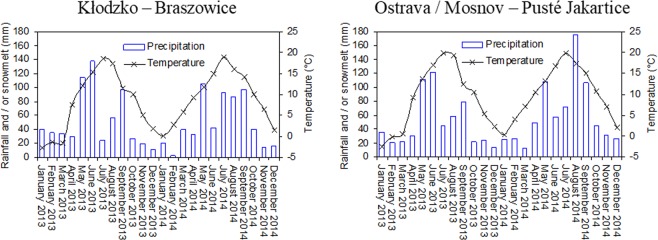
Monthly average temperatures and sums of precipitation in 2013 and 2014 in Kłodzko – Braszowice (Poland) and Ostrava/Mosnov – Pusté Jakartice (Czech Republic).

The average annual long-term air temperature in Braszowice was 8.2 °C and the sum of precipitations was 568.9 mm. The corresponding values in Pusté Jakartice were 8 °C and 640 mm. In Braszowice, the mean annual temperature was lower than the long-term value by 0.4 °C in 2013 and by 1.1 °C in 2014. The annual sum of precipitations in the successive years was higher by about 56 and 20 mm than the long-term average (Table 1), while the sum of precipitation during the growing season (April-September) in both years was similar and amounted to about 460 mm. In Pusté Jakartice, the annual temperatures in 2013 and 2014 were higher than the long-term average by 0.92 and 2.29 °C, respectively. The sum of precipitations was by 58 mm lower in 2013 and by 94 mm higher in 2014 than the long-term average. During the growing season (April-September), the sum of precipitations was greater by 124 mm in 2014 than in 2013. The monthly distribution of the sum of precipitations and average temperature are shown in Fig. 2.

**Table 1:** Annual mean, maximum, and minimum temperatures and sums of precipitations as well as sums of precipitations during the vegetation period (IV–IX) in Kłodzko – Braszowice (Poland) and Ostrava/Mosnov – Pusté Jakartice (Czech Republic).

	T (°C)	Tmax (°C)	Tmin (°C)	Precipitation (mm)	Precipitation IV–IX (mm)
Kłodzko/Braszowice – 2013	7.81	11.93	3.54	624.8	459.1
Kłodzko/Braszowice – 2014	9.30	13.92	4.71	589.3	456.7
Ostrava/Mosnov – 2013	8.92	13.14	4.28	581.6	443.7
Ostrava/Mosnov – 2014	10.29	14.93	5.38	734.1	567.7

## Results

### Exogenous organic materials

The organic carbon and nitrogen contents in the EOMs studied varied from 17.9% in Ra to 40.7% in Dg and from 2.3 in Ra to 8.4% in Mb, respectively. The ranges of variations of the water content and bulk density were 0.046–0.865 m^3^ m^−3^ and 0.117–0.702 Mg m^−3^, respectively^31^. The highest (0.702 Mg m^−3^) and lowest (0.117 Mg m^−3^) bulk density were noted for Mb and Dg, respectively, whereas an opposite result was found for the soil water content, namely the smallest value was exhibited by Mb (0.046 m^3^ m^−3^) and the highest level was determined for Dg (0.865 m^3^ m^−3^). The intermediate values in the case of Ra and Ag for soil organic carbon, Ntot, water content, and bulk density were estimated at 17.9 and 24.1%, 2.3 and 2.6%, 0.258 and 0.225 m^3^ m^−3^, and 0.461and 0.655 Mg m^−3^, respectively.

### Maize yield

The means and the minimum and maximum values of all yield components in both study years, except for straw, in 2013 were greater in Pusté Jakartice than Braszowice (Table 2). The grain yield in Pusté Jakartice in 2013 was higher by 37.5% than in Braszowice (10.07 t ha^−1^), while the straw yield was higher by 15.8% in Braszowice than in Pusté Jakartice (11.34 t ha^−1^). The yield of maize for silage in 2014 was higher in Pusté Jakartice by 16.7% than in Braszowice (16.52 t ha^−1^). Irrespective of the yield component and study year, the CV values ranged from 12.4 to 17.1% in Braszowice and from 7.5 to 9.7% in Pusté Jakartice. The asymmetry (skewness) of the yield components was positive in Braszowice (0.087–0.294), whereas it varied in 2013 from positive 0.162 for the straw yield to negative −0.076 for the grain yield in Pusté Jakartice. The kurtosis ranged from positive 0.418 to negative −0.186 in Braszowice and from positive 0.126 to negative −0.874 in Pusté Jakartice. The skewness and kurtosis values indicate that the yield components were close to the normal distribution.

**Table 2:** Maize yield components in 2013 and 2014 in Braszowice and Pusté Jakartice. (n – number of sample; SD – Standard deviation; CV – Coefficient of variation. The silage dry matter contents were 36.31% in Braszowice and 25.9% in Pusté Jakartice).

Statistics	Grain yield	Grain yield at 14% water content	Straw yield	Grain plus straw yield	Silage yield 2014	Silage yield 2014 (dry mass)
n	40	40	40	40	40	40
**Braszowice (yield, t ha**^**−1**^**)**						
Mean	8.83	10.07	13.13	21.96	45.49	16.52
SD	1.28	1.46	2.24	3.35	5.65	2.05
CV (%)	14.51	14.51	17.09	15.27	12.41	12.41
Minimum	5.64	6.43	8.64	14.28	33.60	12.20
Maximum	11.14	12.70	18.72	29.40	60.61	22.01
Skewnesss	0.087	0.087	0.294	0.121	0.112	0.112
Kurtosis	0.061	0.061	−0.186	−0.143	0.418	0.418
**Pusté Jakartice (yield, t ha**^**−1**^**)**						
Mean	12.15	13.85	11.34	23.48	74.44	19.28
SD	1.10	1.26	1.10	1.75	6.49	1.68
CV (%)	9.07	9.07	9.71	7.47	8.71	8.71
Minimum	10.03	11.43	8.90	19.73	62.99	16.31
Maximum	14.13	16.11	13.75	27.88	87.14	22.57
Skewnesss	−0.076	−0.076	0.289	0.162	0.046	0.046
Kurtosis	−0.874	−0.874	0.126	0.068	−0.857	−0.857

**Figure 3:**
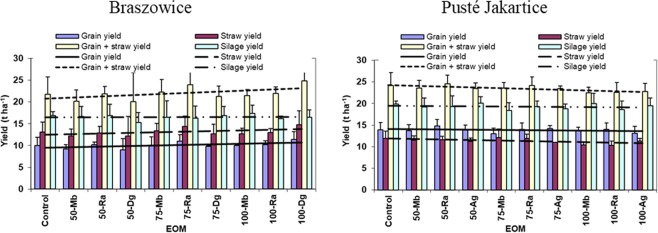
Mean values with standard deviations and linear regression lines between the exogenous organic mater material (EOM) application rates and the grain yield, straw yield, grain plus straw yield in 2013 and the yield of silage maize in 2014 (successively from the left).

The effect of the increasing EOM application rate on the maize yield was related to the study site, year, and yield component (Fig. 3). In Braszowice, the yield of both the maize grain and the straw yield displayed a general ascending trend with the increasing EOM application rate. The most pronounced trend was noted for the combined yield of grain and straw in 2013 and the least distinct trend was found for the silage yield in 2014 when the yield remained almost the same at all EOM types and application rates. In Pusté Jakartice, however, the trends were in general descending, irrespective of the study year and maize yield component.

### Bland-A ltman plots

**Figure 4:**
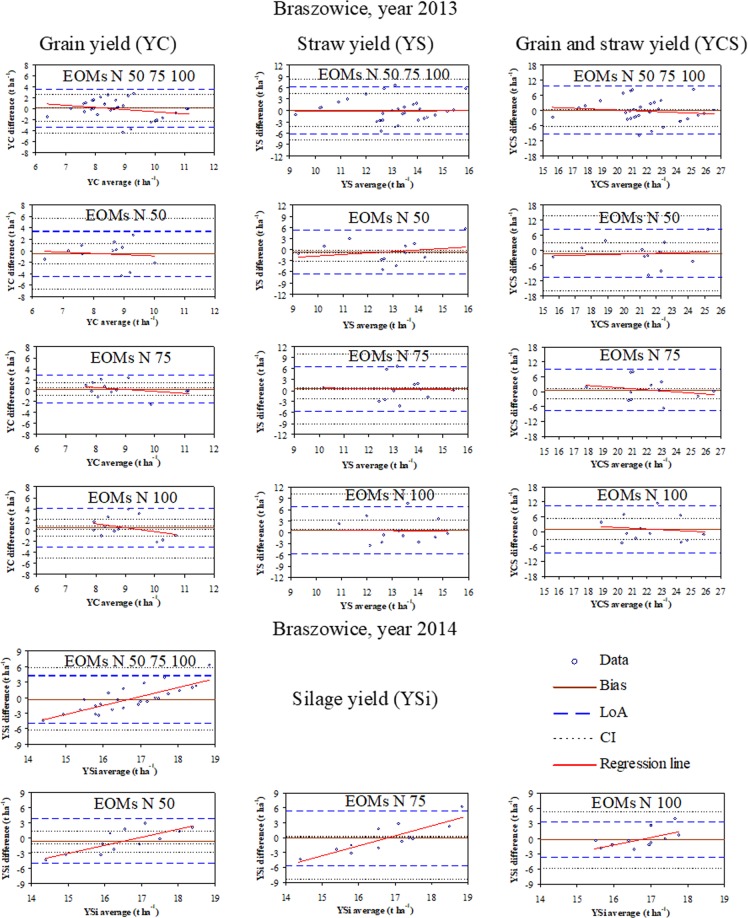
Bland-Altman plots for maize grain, straw, and grain plus straw yields in 2013 and silage yield in 2014 for all application rates of exogenous organic materials (EOMs) and for particular rates. Explanations: EOMs N 50 75 100 = all EOMs application rates as defined further, EOMs N 50 = 50% N from EOMs and 50% mineral N, EOMs N 75 = 75% N from EOMs and 25% mineral N, and EOMs N 100 = 100% N from EOMs, bias line (Bias), limits of agreement (LoA), confidence intervals (CI) for the bias and LoA.

**Figure 5:**
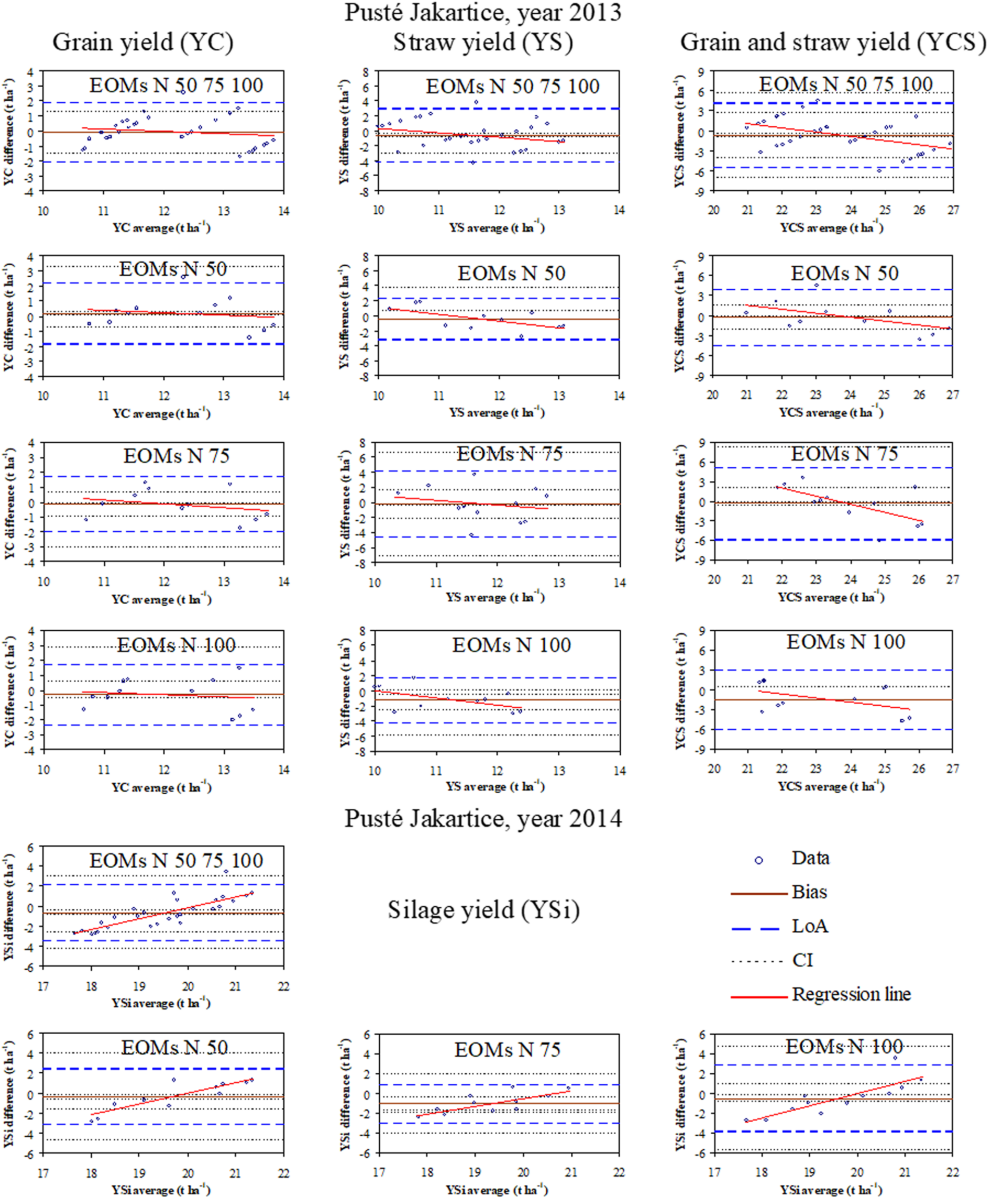
Bland-Altman plots for maize grain, straw, and grain plus straw yields in 2013 and silage yield in 2014 for all application rates of exogenous organic materials (EOMs) and for particular rates. Explanations: EOMs N 50 75 100 = all EOMs application rates as defined further, EOMs N 50 = 50% N from EOMs and 50% mineral N, EOMs N 75 = 75% N from EOMs and 25% mineral N, and EOMs N 100 = 100% N from EOMs, bias line (Bias), limits of agreement (LoA), confidence intervals (CI) for the bias and LoA.

The linear regressions discussed in the section above demonstrate a foremost tendency to change the maize yields in response to the EOM addition. The use of Blant-Altman plots, however, allowed determining the impact of the particular application rates of EOMs on the maize yield. Figures 4 and 5 show horizontal lines of bias, limits of agreement (LoA) along with confidence intervals (CI), and regression lines intersecting or overlapping with bias lines. In general, the values of CI for bias were relatively small and considerably greater with LoAs.

As indicated by the bias values (<0), the application of 50% N from EOMs in Braszowice in 2013 resulted in lower grain, straw, and grain plus straw yields compared to the control (only mineral N) (Fig. 4). Maize yield components increased with the increasing application rates from 75 to 100% N from EOMs (bias >0) and were greater compared to the control. In 2014, as indicated by the bias (<0) lines, the yield of maize for silage increased with the increasing EOM application rate but was smaller on all EOM-amended plots than in the control. In Pusté Jakartice, however, the addition of only 50% N from EOMs increased the grain yield in 2013 (bias >0) in contrast to the other EOM application rates (Fig. 5). The straw and grain plus straw yields decreased with the increasing EOM application rate (bias <0) and were lower than in the control plots. In 2014, the yield of silage maize was lower on the EOM-amended than control plots.

Comparison of Figs. 4 and 5 indicates that the range of limits of agreement (LoA) was from 1.5 to more than 3 times greater for Braszowice than Pusté Jakarice, indicating that the maize yield was more even in the latter. This was confirmed by the similarly larger values of root mean square residuals (RMSR) and maximum relative residuals (MRR) for Braszowice than Pusté Jakartice (Table 3). The Bland Altman ratio value (BAR) indicates that the LoA values were very good or satisfactory (according to^22^) for Pusté Jakartice (0.72–0.153) and in general moderate for Braszowice (0.139–0.236). Hence, the results from Braszowice are burdened with greater uncertainty in 2013 but are much more certain in 2014. The Bland-Altman plots indicate that, regardless of the experimental site and study year, a majority of the points are within the limits of agreement (LoA) and those outlying – within the confidence intervals (CI) (Figs. 4 and 5). The regression line of the yield differences between the EOM-amended and control plots against the average yield of both indicates that the trend in 2013 was only descending in Pusté Jakartice, whereasit was descending, ascending, or almost unchanged (close to bias line) in Braszowice. In 2014, however, the trend was ascending regardless of the EOM application rate and study site. The intersections of the regression lines with the bias lines can be indicative of the yield-producing effectivenes of EOMs. The values of the yield differences above the bias lines, generally in the regions to and from the intersection point of the regression line with the bias line, indicate a greater yield-producing effect of nitrogen from EOMs than from the mineral fertiliser. However, the values below the bias lines indicate a lesser effect of EOMs on the yield than mineral fertilisation. The greater or lesser effect of EOMs on the yield of maize may be related to the type of EOM. This aspect requires further research taking into account the availability and uptake of nitrogen by plants from individual EOMs.

**Table 3:** Root mean square residuals (RMSR), maximum relative residuals (MRR), and Bland-Altman ratio (BAR) of differences in the maize yield between the EOM-amended and control plots.

Statistics	Maize yield (t ha^−1^)			
	Grain	Straw	Grain plus straw	Silage
**Braszowice**				
RMSR (t ha^−1^)	1.804	3.153	4.781	2.388
MRR (%)	53.8	76.8	67.1	40.1
BAR (−)	0.201	0.236	0.214	0.139
**Pusté Jakartice**				
RMSR (t ha^−1^)	1.027	1.912	2.603	1.569
MRR (%)	23.5	38.5	21.8	18.6
BAR (−)	0.082	0.153	0.103	0.072

### Semivariogram models and parameters

The semivariogram spherical models were fitted to the empirical semivariograms for the maize yield (Table 4) with R^2^ from 0.309 to 0.507 for Braszowice and from 0.619 to 0.956 for Pusté Jakartice. The nugget (C0) values for all yield components in Braszowice were relatively low and varied from 0.24 to 0.59 in 2013 and were considerably higher for the yield of silage maize (1.352) in 2014. The corresponding sill values (C0 + Cs) were 1.762–11.89 and 4.181. In Pusté Jakartice, the nugget values for all yield components were 0.116–1.2 in 2013 and 0.550 for the yield of silage maize in 2014 with corresponding sill values 1.228–4.006 and 5.07. The values of the relative structural variance (C0/(C0 + Cs)) for all yield components indicate that the spatial dependence was very strong (<0.25) or moderate (0.25–0.75)^32^. The effective ranges (A) of the spatial dependencies of the yield components in 2013 varied from 17.0 to 18.6 m and from 13.3 to 43.7 m for Braszowice and Pusté Jakartice, respectively. The corresponding effective ranges for the silage maize yield in 2014 were 31.3 and 50.0 m. It is worth noting that the effective ranges were lower in Braszowice than Pusté Jakartice although the plot size was greater in the former. This indicates that the distribution of the yields was more random in Braszowice than Pusté Jakartice.

**Table 4:** Semivariogram models and parameters for the maize yield (t ha^−1^) in 2013 and 2014 in Braszowice and Pusté Jakartice. (Sph. – spherical, C0 – nugget, C0+ C – sill, A – effective range).

Parameters	Model	C0	C0+ C	A (m)	C0/(C0+ C)	R^2^
**Braszowice**						
**Year 2013**						
Grain yield	Sph.	0.367	1.762	18.6	0.208	0.407
Straw yield	Sph.	0.240	5.259	17.0	0.046	0.309
Grain plus straw yield	Sph.	0.590	11.89	17.7	0.050	0.309
**Year 2014**						
Silage yield	Sph.	1.352	4.181	31.3	0.324	0.507
**Pusté Jakartice**						
**Year 2013**						
Grain yield	Sph.	0.116	1.970	43.7	0.059	0.941
Straw yield	Sph.	0.589	1.228	13.3	0.480	0.619
Grain plus straw yield	Sph.	1.200	4.006	31.3	0.300	0.895
**Year 2014**						
Silage yield	Sph.	0.550	5.070	50.0	0.108	0.956

### Maps of maize yield

**Figure 6:**
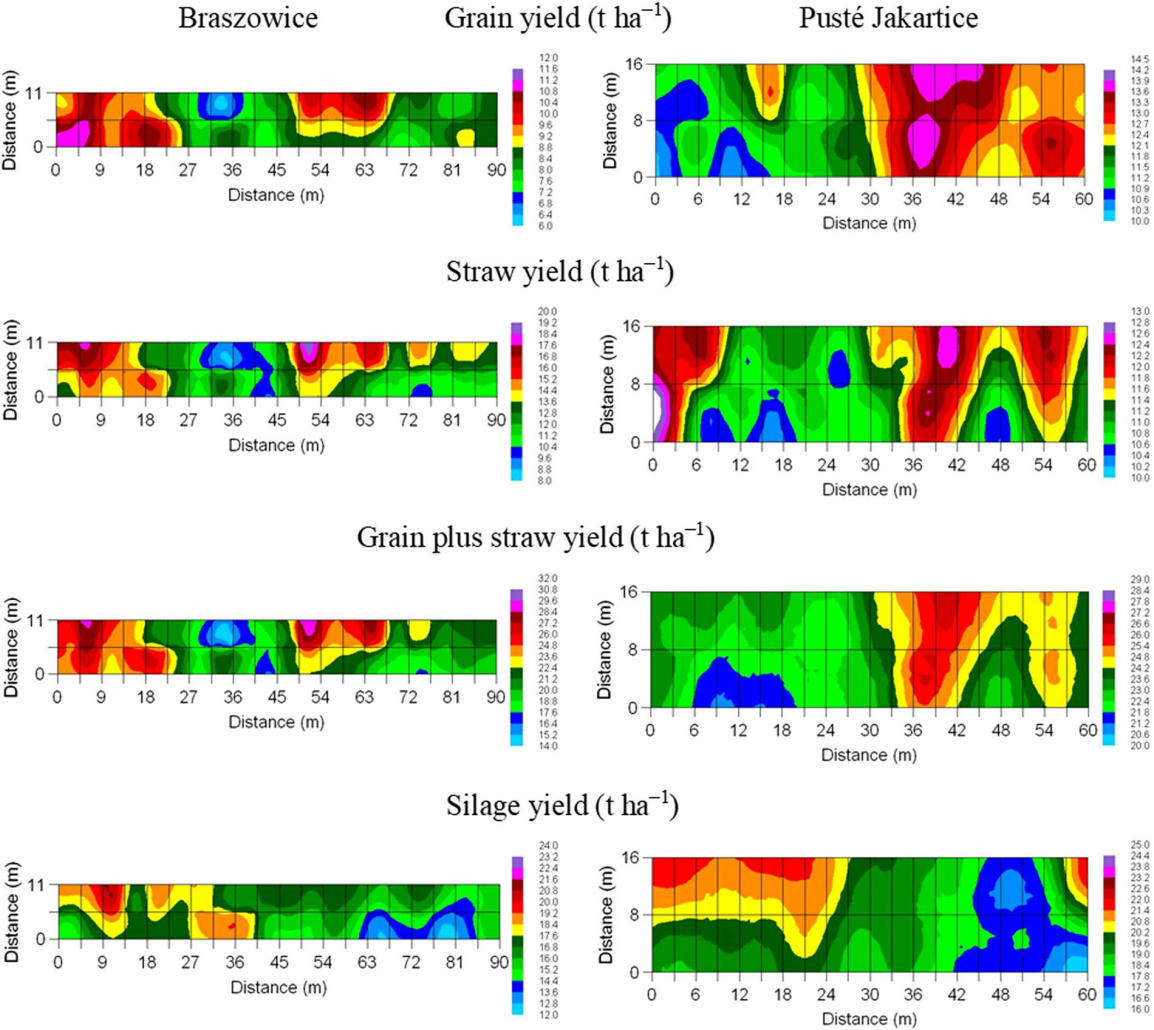
Spatial distribution of maize yield in 2013 and 2014 in Braszowice and Pusté Jakartice.

As can be seen in Fig. 6, two distinct zones can be separated with similar yield responses in both sites corresponding approximately to half the field length. These zones are from 0 to 45 m and 45 to 90 m in Braszowice and from 0 to 30 m and 30 to 60 m in Pusté Jakartice. Such separation can be related to the random distribution of plots fertilised with different types and application rates of EOMs, which was the same in both sites (Fig. 1). It is worth noting that the concentration of plots with the large doses of EOMs in some areas influences the crop yield differently in particular sites. For example, the impact of 3 out of the 4 plots with a 100% N from EOMs in the lower part of the field on the maize yield is positive from 9 to 27 m in Braszowice and negative from 6 to 18 m in Pusté Jakartice. Another example is the smaller maize yield in the area with greater N levels from EOMs from 42 to 54 m in the lower part of the field both in 2013 and 2014 in Pusté Jakartice. In turn, a larger yield was noted in the area from 18–30 m in the lower part in Pusté Jakartice, where plots with a dose of 50% N from EOMs dominated (Fig. 1). Mapping of maize yield can support variable application of nitrogen from EOMs across the field.

## Discussion

The regression lines (trends) showed that the maize yield tended to increase and decrease with the increasing EOM application rate in Braszowice and Pusté Jakartice, respectively although the absolute yield was greater in the latter (Fig. 2). This tendency was less pronounced in Pusté Jakartice. The greater and less variable absolute maize yield in Pusté Jakartice than Braszowice can be related to the greater soil water content at most occasions, as reported in the accompanying paper^31^ and thereby better water supply for maize. These differences in the soil water content can be attributed in part to the greater water holding capacity in Pusté Jakartice (33.82 m^3^ m^–3^) than Braszowice (31.80 m^3^ m^–3^)^26^ as a result of better soil aggregation and the presence of a greater number of earthworms that we observed during the field measurements. Both stabilize the pore structure and permit storage of greater amounts of water in soil profile. Futhermore, more variable maize yield in the less productive Braszowice than Pusté Jakartice soil can in part result from its greater sensitivity to changes in soil water content associated with EOM application.

The study allowed identifying the specific benefits of three statistical approaches for description of maize yield response to different levels of exogenous organic matterials. Classical statistics allowed to recognize the general trends of the impact of exogenous organic matterials on yield based on mean values, dispersion, variability and asymmetries in distribution and kurtosis. However, the use of geostatistical methods including semivariograms and kriging-interpolated maps permitted delineating low-yielding sub-field areas on two soils of different agricultural suitability under variable application of oxogenous organic matters. The Bland-Altman plot analysis showed for the first time in what manner the partial and total replacement of readily available nitrogen from the synthetic fertiliser with N from exogenous organic materials affected the maize yield in particular sites. In Braszowice, a positive increase in the grain yield occurred only after the application of the larger doses of organic N (75 and 100% N from EOMs). In turn, in the Pusté Jakartice soil, this was observed already after the addition of the smallest dose, i.e. 50% N from EOMs. The less sensitive grain yield response to organic N in Braszowice vs. Pusté Jakartice can be elucidated by the visually observed less friable structure in the former that diminishes the ability of roots to grow and uptake water and nutrients^33^. The more efficient yield-producing effect of organic N in Braszowice than Pusté Jakartice was also observed with the yield of silage maize. The different maize yield response to the EOM application in Braszowice and Pusté Jakartice revealed by the Bland-Altman statistics is in line with the spatial yield distribution on the kriging maps. The maps showed that, in areas with dominance of plots amended with 100% N from the EOMs, the grain yield was larger in Braszowice and smaller in Pusté Jakartice compared to the neighboring areas with the majority of plots amended with 50% N from EOMs. These results are of practical importance, as they indicate that more organic N is required in the less productive soil with poorer structure in Braszowice than Pusté Jakartice to increase the maize grain yield. They will help to develop management strategy to reduce losses of the unused by crops nitrogen through volatilization and leaching into the environment as well as farm input costs depending on environmental conditions. Recent studies (e.g.^33,34^) have shown that nitrogen uptake and utilisation efficiency in maize can be influenced by tillage systems.

Review of the literature indicates that the effect of soil amendment with organic matter should be considered not only with respect to crop production but together with the broader ecosystem services and benefits, including carbon sequestration^7,18,35^, climate change mitigation and adaptation^3,9^, improvement of resistance to soil erosion and soil compaction^3,36,37^, and better natural pest control^4^, which lead to reduced reliance on mineral N fertilisers or other farm chemicals and lower environmental pollution^4,38^. Moreover, biological research on the same fields as those used in this study showed that the addition of EOMs stimulated the activity of dehydrogenases and the poorly explored microbial functional and genetic diversity in soil^39^. It also demonstrated that EOMs are not a threat to the soil edaphone^40^ and concentration of carbon dioxide in the atmosphere^41,42^. Given the yield response observed in the present study and the ecosystem benefits, precise application of the exogenous organic materials offers an opportunity for sustainable crop production and environmental protection as well as proper management of organic wastes from agricultural and biogas production systems.

The Bland-Altman plots also demonstrate that the regression lines of maize yield in 2013 were close to the bias lines in both sites, while they increased with the increasing average yield from the EOM-amended and control plots in 2014. These interannual differences may be related to the relatively high sum of precipitations in 2013 during intensive maize growth (May-June) than in the other period and during the later growth (July-September) in 2014 in both places. This difference may have caused diversification of the spatial distribution of maize yields through different spatial storage and redistribution of water depending on the EOM application rate. Furthermore, the range of the limits of agreement (±1.96 SD lines) of the Bland-Altman plots for both the grain and straw yield is about two times higher for Braszowice than Pusté Jakartice in both study years. This proves that the soil in Pusté Jakartice creates more even growth conditions for plants than that in Braszowice. However, the soil in Braszowice requires optimised and localised treatments to improve and smooth plant growth conditions. Further studies are intended to assess the most yield-producing EOM rates under range of soil and weather conditions and types of exogenous materials using the statistical methodology verified in this work.

## Summary and Conclusions

The yield of maize in response to the application of four exogenous (recycled) organic materials (EOMs) at three application rates was explored and assessed by means of linear regressions (trends), spatial kriging-interpolated maps, and Bland-Altman statistics in two experiments. The regression trends of maize yield in general increased with the increasing EOM application rate in Braszowice and decreased in the more productive Pusté Jakartice soil. The spatial kriging-interpolated maps allowed delineating zones of lower and higher maize yields that were related to the spatial distribution of the EOM application rates. The concentrations of plots with the large doses of EOMs were in general reflected in the increased yield in Braszowice and the reduced yield in Pusté Jakartice. The analysis of the Bland-Altaman statistics revealed that, in 2013, the effect of 50% N from EOMs on the maize yield was negative and positive in Braszowice and Pusté Jakartice, respectively, whereas the inverse was true for the additions of 75 and 100% N from EOMs. However, in 2014 characterised by a greater sum of precipitations during the growing season, the increasing EOM application rate caused an increase and decrease in the maize silage yield compared to the control plots in Braszowice and Pusté Jakartice, respectively. The limits of agreement showed that the maize yield was more variable in Braszowice than in Pusté Jakartice. The study confirmed the hypothesis that the use of the Bland-Altman method allows determining separate effects of various EOMs application rates on the maize yield compared to the maize yield grown on plots fertilised only with mineral nitrogen. The results will support precise adjusting the most yield-producing nitrogen rates from EOMs depending on the study site conditions and weather conditions prevailing during growing season.

## References

[1] Buttafuoco G, Castrignanò A, Cucci G, Lacolla G, Lucà F (2017). Geostatistical modelling of within-field soil and yield variability for management zones delineation: a case study in a durum wheat field. Precision Agriculture.

[2] Cherlet, M. *et al*. World Atlas of Desertification 3rd edition, http://wad.jrc.ec.europa.eu/ (2018).

[3] EASAC (European Academies’ Science Advisory Council). EASAC Opportunities for soil sustainability in Europe. *Policy report* 36, available at, www.easac.eu (2018).

[4] Garratt MPD (2018). Enhancing soil organic matter as a route to the ecological intensification of European arable systems. Ecosystems.

[5] Murty D, Kirschbaum MUF, McMurtrie RE, McGilvray H (2002). Does conversion of forest to agricultural land changes soil carbon and nitrogen? A review of the literature. Glob Change Bio.

[6] Moreno, F., Murillo, J. M. & Madejón, E. Carbon losses under dryland conditions, tillage effects. eds. Gliński, J., Horabik, J. & Lipiec, J. *Encyclopedia of Agrophysics*, Springer, Dordrecht, Heidelberg, London, New York, 108–109 (2011).

[7] Lal R (2004). Soil carbon sequestration impacts on global climate change and food security. Science.

[8] Paustian K (2016). Climate-smart soils. Nature.

[9] Minasny B (2017). Soil carbon 4 per mille. Geoderma.

[10] Post WM, Kwon KC (2000). Soil carbon sequestration and land use change: processes and potential. Glob Change Biol.

[11] Griscom BW (2017). Natural Climate Solutions. Proceedings of the National Academy of Sciences.

[12] Olivier, J. G. J., Janssens-Maenhout, G., Muntean, M. & Peters, J. A. H. W. Trends in global CO_2_ emissions: 2015 Report. PBL Netherlands Environmental Assessment Agency, The Hague, PBL publication number: 1803, *JRC Technical Note number*: JRC98184, http://edgar.jrc.ec.europa.eu/news_docs/pp, 80 (2015).

[13] Zdruli, P., Lal, R., Cherlet, M. & Kapur, S. New World Atlas of Desertification and Issues of Carbon Sequestration, Organic Carbon Stocks, Nutrient Depletion and Implications for Food Security. In Carbon Management, Technologies, and Trends in Mediterranean Ecosystems. Springer International Publishing, 13–25 (2017).

[14] COM/2006/0231 Final. Communication from the Commission to the Council, the European Parliament, the European Economic and Social Committee and the Committee of the Regions: Thematic Strategy for Soil Protection. Commission of the European Communities, http://eur-lex.europa.eu/LexUriServ/LexUriServ.do?uri=COM:2006:0231:FIN:EN:PDF.

[15] Chabbi A (2017). Aligning agriculture and climate policy. Nat Clim Change.

[16] Soussana JF (2017). Matching policy and science: Rationale for the ‘4 per 1000– soils for food security and climate’ initiative. Soil Till Res.

[17] Arnó J, Rosell JR, Blanco R, Ramos MC, Martinez-Casasnovas JA (2012). Spatial variability in grape yield and quality influenced by soil and crop nutrition characteristics. Precision Agriculture.

[18] Kaczyński, R. & Siebielec, G. The influence of exogenous organic matter on the content and quality of soil organic matter. eds. Malý, S. & Siebielec, G. *Testing of Exogenous Organic Materials for Safe Application to the Soil*, Brno, Central Institute for Supervising and Testing in Agriculture, Brno, Czech 31–38, (in Czech) (2015).

[19] Diacono M, Montemurro F (2010). Long-term effects of organic amendments on soil fertility. A review. Agron Sustain Dev.

[20] Lechenet M, Dessaint F, Py G, Makowski D, Munier-Jolain N (2017). Reducing pesticide use while preserving crop productivity and profitability on arable farms. Nature Plants.

[21] Hijbeek R (2017). Do organic inputs matter – a meta analysis of additional yield effects for arable crops in Europe. Plant Soil.

[22] Giavarina D (2015). Understanding Bland Altman analysis. Biochemia Medica.

[23] Usowicz B, Marczewski W, Usowicz JB, Łukowski M, Lipiec J (2014). Comparison of surface soil moisture from SMOS satellite and ground measurements. Int Agrophys.

[24] Zawadzki J, Kędzior M (2016). Soil moisture variability over Odra watershed: Comparison between SMOS and GLDAS data. Int J Appl Earth Obs.

[25] Malý, S. Exogenous organic matter in relation to soil organic matter and ecosystem function. eds. Malý, S. & Siebielec, G., *Testing of Exogenous Organic Materials for Safe Application to the Soil*, Central Institute for Supervising and Testing in Agriculture, Brno, Czech 5–13, (in Polish) (2015).

[26] Lipiec, J., Turski, M., Bieganowski, A. & Usowicz, B. Effect of organic matter addition on soil physical properties. eds. Malý, S. & Siebielec, G., *Testing of Exogenous Organic Materials for Safe Application to the Soil*, Central Institute for Supervising and Testing in Agriculture, Brno, Czech 67–80, (in Polish) (2015).

[27] Niedźwiecki, J., Smatanova, M., Gałązka, R., Cyganek, K. & Siebielec, G. Research methodology and characteristics of investigated organic materials. eds Malý, S. &. Siebielec, G., *Testing of Exogenous Organic Materials for Safe Application to the Soil*, Central Institute for Supervising and Testing in Agriculture, Brno, Czech, 15–29, (in Polish) (2015).

[28] Gamma Design Software (GS + 9). Geostatistics for the environmental sciences (2008).

[29] Zawadzki J, Cieszewski CJ, Zasada M, Lowe RC (2005). Applying geostatistics for investigations of forest ecosystems using remote sensing imagery. Silva Fenn.

[30] Bland JM, Altman DG (1986). Statistical method for assessing agreement between two methods of clinical measurement. The Lancet.

[31] Usowicz, B. & Lipiec, J. The effect of exogenous organic matter on the thermal properties of tilled soils in Poland and the Czech Republic. *J Soil and Sediment*, 10.1007/s11368-019-02388-2 (2019).

[32] Cambardella CA (1994). Field-scale variability of soil properties in Central Iowa soils. Soil Sci Soc Am J.

[33] Ozpinar S (2016). Nutrient concentration and yield of maize (*Zea mays* L.) after vetch (*Vicia sativa* L.) in conventional and reduced tillage systems. J Plant Nutr.

[34] Jug D (2019). Effect of conservation tillage on crop productivity and nitrogen use efficiency. Soil Till Res.

[35] Długosz J, Piotrowska-Długosz A (2016). Spatial variability of soil nitrogen forms and the activity of N-cycle enzymes. Plant Soil Environ.

[36] Batey T (2009). Soil compaction and soil management – a review. Soil Use Manage.

[37] Reichert JM, Suzuki LEAS, Reinert DJ, Horn R, Håkansson I (2009). Reference bulk density and critical degree-of-compactness for no-till crop production in subtropical highly weathered soils. Soil Till Res.

[38] Plaza C (2016). Response of different soil organic matter pools to biochar and organic fertilizers. Agr Ecosys Environ.

[39] Frąc, M. *et al*. Effect of exogenous organic matter (EOM) on functional and genetic diversity of microorganisms and soil enzymatic activity in relation to environmental properties. eds. Malý, S. & Siebielec, G. *Testing of Exogenous Organic Materials for Safe Application to the Soil*, Central Institute for Supervising and Testing in Agriculture, Brno, Czech 81–103, (in Polish) (2015).

[40] Tuf, I. H., & Horňák, O. The response of soil fauna to the exogenous organic matter application for soil fertilisation. eds. Malý, S. & Siebielec, G. *Testing of Exogenous Organic Materials for Safe Application to the Soil*, Central Institute for Supervising and Testing in Agriculture, Brno, Czech 105–114, (in Polish) (2015).

[41] Čuhel, J., & Brzezińska, M. The risk of increasing greenhouse gas emissions from the soil as a result of the use of exogenous organic matter, eds. Malý, S. & Siebielec, G. *Testing of Exogenous Organic Materials for Safe Application to the Soil*, Central Institute for Supervising and Testing in Agriculture, Brno, Czech 115–128, (in Polish) (2015).

[42] Czubaszek R, Wysocka-Czubaszek A (2018). Emissions of carbon dioxide and methane from fields fertilized with digestate from an agricultural biogas plant. Intl Agrophys.

